# Mindfulness Intervention for Navigating and Decreasing Stress (MINDS-V): Effectiveness of a Tailored Trauma-Informed Mental Health Intervention for Australian Veterans

**DOI:** 10.1093/milmed/usaf393

**Published:** 2025-08-06

**Authors:** Wendy Wen Li, Marc Chao, Jennifer Gaskin, Carolyn Heward, Rhein Frank, Timothy Leow

**Affiliations:** Department of Psychology, College of Healthcare Sciences, James Cook University, Townsville, QLD 4811, Australia; Department of Psychology, College of Healthcare Sciences, James Cook University, Townsville, QLD 4811, Australia; Department of Psychology, College of Healthcare Sciences, James Cook University, Townsville, QLD 4811, Australia; Department of Psychology, College of Healthcare Sciences, James Cook University, Townsville, QLD 4811, Australia; 1 Million Strong Townsville Gym, Townsville, QLD 4814, Australia; Mental Health Services, Townsville University Hospital, Townsville, QLD 4814, Australia

## Abstract

**Introduction:**

Veterans face unique mental health challenges, including elevated rates of post-traumatic stress disorder (PTSD), depression, and anxiety, compounded by cultural barriers to traditional therapies. Mindfulness-Based Stress Reduction (MBSR) offers a trauma-informed, non-pharmacological intervention tailored to align with military values.

**Materials and Methods:**

This study employed a repeated-measures design with 4 time points (pre-intervention, mid-intervention, post-intervention, and 2-month follow-up) to evaluate the effectiveness of an MBSR program on Australian veterans’ PTSD, depression, anxiety, and mindfulness outcomes.

**Results:**

Findings revealed significant reductions in PTSD, depression, and anxiety symptoms, but not for mindfulness state. Despite a slight symptom resurgence at follow-up, scores remained significantly improved from baseline.

**Conclusions:**

The tailored MBSR intervention demonstrated significant benefits in managing PTSD, depression, and anxiety. Although changes in mindfulness state were not observed, symptom improvements suggest that mindfulness practice may support emotional regulation. Long-term engagement strategies, such as booster sessions or community-based support groups, are essential for sustaining these outcomes.

## INTRODUCTION

Military service is inherently distinct, placing individuals under intense physical, mental, and emotional pressure.^1^ Although military culture fosters resilience, pride, and belonging, certain values such as self-reliance, masculine honor, and conformity to traditional norms may contribute to poor mental health outcomes and deter help-seeking behavior.^1^ Globally, veterans experience higher rates of mental health disorders, including post-traumatic stress disorder (PTSD), depression, anxiety, and substance misuse, compared to civilians.^2^

In Australia, a veteran broadly refers to anyone who has served and is currently serving in the Australian Defence Force (ADF).^3^ To align with terminology used in international studies, the term “veteran” in the current study refers specifically to ex-serving members of ADF. Mental health conditions among Australian veterans remain a critical concern.^4^ Compared to the general Australian population, military personnel face unique stressors, including exposure to combat, frequent relocations, and prolonged family separations, often leading to physical and mental trauma.^1^ Post-traumatic stress disorder and depression rates are notably higher among ADF veterans than the general population, with nearly half developing a mental disorder within 5 years of discharge.^5^ Post-traumatic stress disorder and anxiety are most prevalent, with 24.9% meeting lifetime PTSD criteria and 17.7% experiencing it in the past year.^5^

International guidelines emphasize trauma-focused therapies (TFTs), including cognitive-behavioral therapy (CBT), prolonged exposure therapy, and Eye Movement Desensitization and Reprocessing (EMDR), which target trauma-related thoughts, emotions, and avoidance behaviors.^6^ However, barriers such as adverse side effects including nightmares, flashbacks, increased substance use, emotional unpreparedness, and perceived treatment ineffectiveness often hinder participation in TFTs.^7^ Treatment dropout rates among veterans range from 5% to 78%, averaging 36%—significantly higher than in civilian populations.^8^ Ambivalence about confronting traumatic memories and a preference for non-trauma-focused approaches are key factors driving this attrition.^9^

Systemic barriers, including mistrust of clinical services and limitations of one-on-one therapy models, further highlight the need for alternative interventions tailored to veterans’ preferences.^10^ Widely studied group-based, non-trauma-focused therapies such as Mindfulness-Based Stress Reduction (MBSR) present a promising alternative to TFTs.^11^ Mindfulness refers to “the awareness that emerges through paying attention on purpose, in the present moment, and nonjudgmentally to the unfolding of experience moment by moment.”^12^ In this definition, mindfulness is regarded as a state of detached observation and present-moment awareness. It can be developed through mindfulness meditation, which fosters greater awareness of internal experiences (such as thoughts and emotions) as they arise, while maintaining a non-judgmental attitude.^12^^,^^13^ Mindfulness meditation promotes emotional regulation by helping individuals respond thoughtfully to stressful situations rather than reacting automatically,^13^^,^^14^ in turn reducing emotional reactivity and building resilience.^15^^,^^16^

Meta-analyses examining the effectiveness of MBSR programs have demonstrated significant reductions in PTSD and depression symptoms among veterans, with medium effect sizes compared to control groups.^13^ Increases in mindfulness have also been found among military veterans following MBSR programs, both immediately after the intervention and at follow-up.^13^ Mindfulness-based stress reduction programs have been shown to foster self-compassion, reduce self-stigma, and align with military values of discipline, focus, and perseverance, making them a culturally congruent therapeutic approach.^13^ Specifically, MBSR promotes discipline through regular mindfulness meditation, enhances focus by training attention on the present moment, and builds perseverance by encouraging consistent effort and resilience in the face of stress.^14^ Mindfulness-Based Stress Reductions structured 8-week format, featuring group sessions, guided meditation, and homework, mirrors military training routines, offering familiarity and consistency.^17^,^18^ This structure supports post-­service adjustment by fostering stability, purpose, and identity.^19^,^20^ The group setting also promotes camaraderie, reduces isolation, and enhances engagement through mutual accountability.^4^ Moreover, MBSR offers a non-pharmacological alternative for managing anxiety, producing effects comparable to medications like escitalopram.^21^

The concept of trauma-informed care is also increasingly recognized as crucial in delivering mental health interventions for individuals with a history of trauma and PTSD symptoms, including military veterans.^21^ It is recommended that interventions consider how past trauma and life stressors might impact an individual’s current life,^21^^,^^22^ with the primary goal of minimizing the risk of re-traumatization during treatment.^13^ Trauma-­Informed Mindfulness Based Stress Reduction (TI-MBSR) is a model designed to enhance the therapeutic benefits of MBSR for those with a history of trauma. Trauma-Informed Mindfulness Based Stress Reduction emphasizes the principals of trauma-­informed approaches, including safety, trustworthiness empowerment, and choice, and incorporates grounding and resourcing techniques to aid in self-regulation.^22^ Additionally, TI-MBSR includes the core experiential practices of standard MBSR while integrating trauma-specific psychoeducation on topics such as the neurophysiology of stress and trauma, symptoms as adaptive responses, and the interplay of cognitive and survival thinking.^22^ By providing a trauma-informed framework, TI-MBSR aims to be a more suitable and effective intervention for trauma survivors. For veterans hesitant about exposure-based therapies, TI-MBSR offers a safer alternative, enabling them to address avoidance behaviors and develop emotional flexibility without directly confronting traumatic memories.^23^ Participation in TI-MBSR can foster reduced emotional reactivity and improved coping strategies,^22^ supporting veterans to engage meaningfully in trauma-focused therapies in the future and further enhance their mental well-being.

This study evaluates the effectiveness of a tailored TI-MBSR intervention for ADF veterans, specifically its impact on symptoms of PTSD, depression, anxiety, and mindfulness. Using a repeated-measures design across 4 time points, it assesses longitudinal symptom changes. The study hypothesizes significant reductions in PTSD symptoms—including re-­experiencing, avoidance, negative cognition and mood, and hyperarousal—alongside decreases in depression and anxiety. Additionally, mindfulness levels are expected to increase, reflecting improved emotional regulation and present-moment awareness.

## METHODS

### Research Design

This study used a single-arm, repeated-measures design with 4 measurement points: pre-intervention (T1), mid-intervention (T2), post-intervention (T3), and 2-month follow-up (T4).

### The Intervention: Tailored MBSR

The Trauma-Informed Mindfulness Based Stress Reduction intervention, delivered from February 2023 to May 2024, followed the Brown University Mindfulness Centre’s MBSR Curriculum and Teaching Guide.^24^ Conducted by a certified MBSR teacher, sessions ran weekly for 2.5 hours over 8 weeks, plus a 2-hour orientation. A psychiatrist was available on-site but was not required during the study. Participants received home-practice materials and daily guided meditation recordings (20-30 minutes, 6 days a week).

Although retaining the core principles of the standardized MBSR framework, tailored adaptions were made to address the unique experiences and challenges faced by veterans by adopting a trauma-informed approach. These modifications were modelled on other studies employing mindfulness-based interventions with participants with a history of trauma,^21^^,^^22^ as well as military veterans,^4^^,^^9^^,^^23^ and recommendations by the Commonwealth of Australia^25^ for working with veterans. An outline of the modifications is presented in **Table S1**.

### Participants

Eligible participants were ex-ADF members discharged before the commence of the intervention, aged 18-65, and fluent in English. Exclusion criteria included current diagnosis of substance use disorders, psychotic disorders, severe cognitive impairment, suicidal ideation, recent medication changes, unstable medical conditions, ongoing psychological therapies, or prior MBSR experience.

Participants were recruited through community organizations, gyms, and Facebook in a regional Australian city using convenience sampling methods. Forty-one participants (forming 5 groups) completed the pretest (T1) questionnaire, and 34 completed Week 1 of the MBSR program. Of the 7 participants who withdrew before Week 1, one withdrawing because of work commitments, 4 because of time constraints, and 2 because of lack of interest. At in-session test (T2), 30 participants remained, with dropouts because of family (*n *= 3) and work (*n *= 1) commitments. At post-test (T3), 28 participants remained, with one withdrawing from the study because of enrolling in a trauma therapy, and another leaving because of work commitments. Twenty-three participants completed the follow-up questionnaire (T4), with 5 lost to contact. Participant characteristics are detailed in **Table 1**. The rates of PTSD, depression, and anxiety at T1 are presented in **Table 2**.

**Table 1.** Participant Characteristics

Variable	Category/statistic	*N*, M (SD), %
Participants	Total	*N* = 41
Age	Mean (SD)	46.17 (11.77)
Annual income	Mean (SD)	$70,018 ($23,266)
Number of deployments	Mean (SD)	2.08 (1.8)
Deployment lengths (months)	Mean (SD)	14.61 (17.09)
Time since discharge (months)	Mean (SD)	134.85 (141.84)
Length of prior mindfulness practice (years)	Mean (SD)	4.55 (5.35)
Gender	Male	92.70%
Marital status	Married	48.80%
	Separated	12.20%
	Divorced	26.80%
	Never married	9.80%
	Not reported	2.40%
First nations identification	Yes	4.90%
Employment status	Employed	17.10%
	Unemployed	80.50%
	Not reported	2.40%
Educational attainment	Bachelor’s degree or higher	14.60%
	Technical and Further Education (TAFE) or other post-secondary certificates	58.50%
	High school	22%
	Did not complete high school	4.90%
Financial support	Disability pension	39%
	Veteran service pension	22%
	Income support supplement	9.80%
	Incapacity payments	7.30%
	Not reported	22%
Living arrangements	Lived alone	17.10%
	Couple relationship without children	24.40%
	Couple relationship with children	34.10%
	Single parent	14.60%
	Not reported	2.40%
Disability services	On disability services	31.70%
Veteran support services	On veteran support services	68.30%
Smoking status	Smokers	19.50%
Mindfulness practice	Engaged in mindfulness practice	56.10%
Type of mindfulness practice	Formal mindfulness practice	26.80%
	Informal mindfulness practice	29.30%
	Not reported	43.90%
Psychotropic medications	Currently on medications	61%
Psychological treatment	Currently receiving treatment	73.20%

Abbreviations: *N*, total sample size; M, mean; SD, standard deviation.

**Table 2.** Rates of PTSD, Depression, and Anxiety at T1

	Cutoff scores	Severity	*N*	%
T1 PTSD	0-32	Normal	16	39
	≥33	Elevated	25	61
T1 depression	0-4	None-minimal	7	17.1
	5-9	Mild	7	17.1
	10-14	Moderate	9	22.0
	15-19	Moderately severe	13	31.7
	≥20	Severe	5	12.2
T1 anxiety	0-4	None-minimal	6	14.6
	5-9	Mild	14	34.1
	10-14	Moderate	11	26.8
	≥15	Severe	10	24.4

Abbreviations: *n*, sample size; PTSD, post-traumatic stress disorder; T1, time point 1 or baseline; total sample size at baseline = 41.

### Measures

#### Post-traumatic stress disorder

Assessed using the PTSD Checklist for DSM-5 (PCL-5),^26^ a 20-item self-report scale measuring symptom severity. Scores range from 0 to 80, with higher scores indicating greater severity. The scale includes 4 subscales: re-experiencing (PTSD-R), avoidance (PTSD-A), negative alterations in cognition and mood (PTSD-N), and hyperarousal (PTSD-H). Internal consistency across time points was excellent (Cronbach’s alpha: 0.96-0.97).

#### Depression

Measured using the 9-item Patient Health Questionnaire (PHQ-9),^27^ a validated self-report tool assessing symptom severity over the past 2 weeks. Scores range from 0 to 27, with higher scores indicating greater severity. Internal consistency ranged from 0.89 to 0.92 across time points.

#### Anxiety

Evaluated using the 7-item Generalized Anxiety Disorder Scale (GAD-7),^28^ assessing symptom severity over the past 2 weeks. Scores range from 0 to 21, with higher scores reflecting greater anxiety. Internal consistency ranged from 0.91 to 0.95.

#### Mindfulness

Measured using the 15-item Mindful Attention Awareness Scale (MAAS),^29^ assessing the frequency of mindful awareness in daily life. Scores range from 1 to 6, with higher scores indicating greater mindfulness. Internal consistency ranged from 0.83 to 0.92.

### Procedure

Ethics approval was obtained from the Human Research Ethics Committees of the Australian Government Departments of Defence and Veterans’ Affairs (Ref. 2022/BN45322893), Townsville University Hospital (Blinded for peer review; Ref. HREC/2022/QTHS/84532), and James Cook University (Ref. H8787). Participants registered via QR code and received an information package before orientation.

Upon consent, research assistants (RAs) collected paper-based questionnaires and assigned unique ID numbers for confidentiality. Data were de-identified before analysis. Assessments occurred at T1, T2, T3, and T4.

### Data Analysis

Data were analyzed using IBM SPSS Statistics v29. Outliers were identified using *z*-scores and visually inspected with box plots; mean substitution was applied to one outlier on the MAAS scale at T3. Normality was assessed using the Shapiro-Wilk test, with some subscales showing *P*-values <.05. However, Q-Q plot visual inspections indicated approximate normality; nevertheless, given that the Shapiro-Wilk test results did not consistently support normality across all variables, non-parametric analyses were conducted. The Shapiro-Wilk scores and Q-Q plots are presented in **Table S3** and **Figure S1**, respectively.

Given the non-normal distribution of several variables, non-parametric analyses were conducted. The Friedman test was used to assess overall changes in scores across 4 time points (T1, T2, T3, and T4). Where significant omnibus effects were observed, post-hoc pairwise comparisons were performed using Wilcoxon signed-rank tests. A Bonferroni-adjusted alpha level of 0.017 was applied to control for multiple comparisons. Effect sizes for Wilcoxon tests were calculated using the formula *r *=* Z*/√*N*, where *N* represents the number of non-tied observations, and *r* values of approximately 0.10, 0.30, and 0.50 were interpreted as small, moderate, and large effects, respectively.

## RESULTS

### Preliminary Analysis

Means, SDs, and intercorrelations among all outcome variables at baseline and weeks 4, 8, and 12 are presented in **Table S2**.

### Effectiveness of the MBSR Intervention

**Figure 1.**
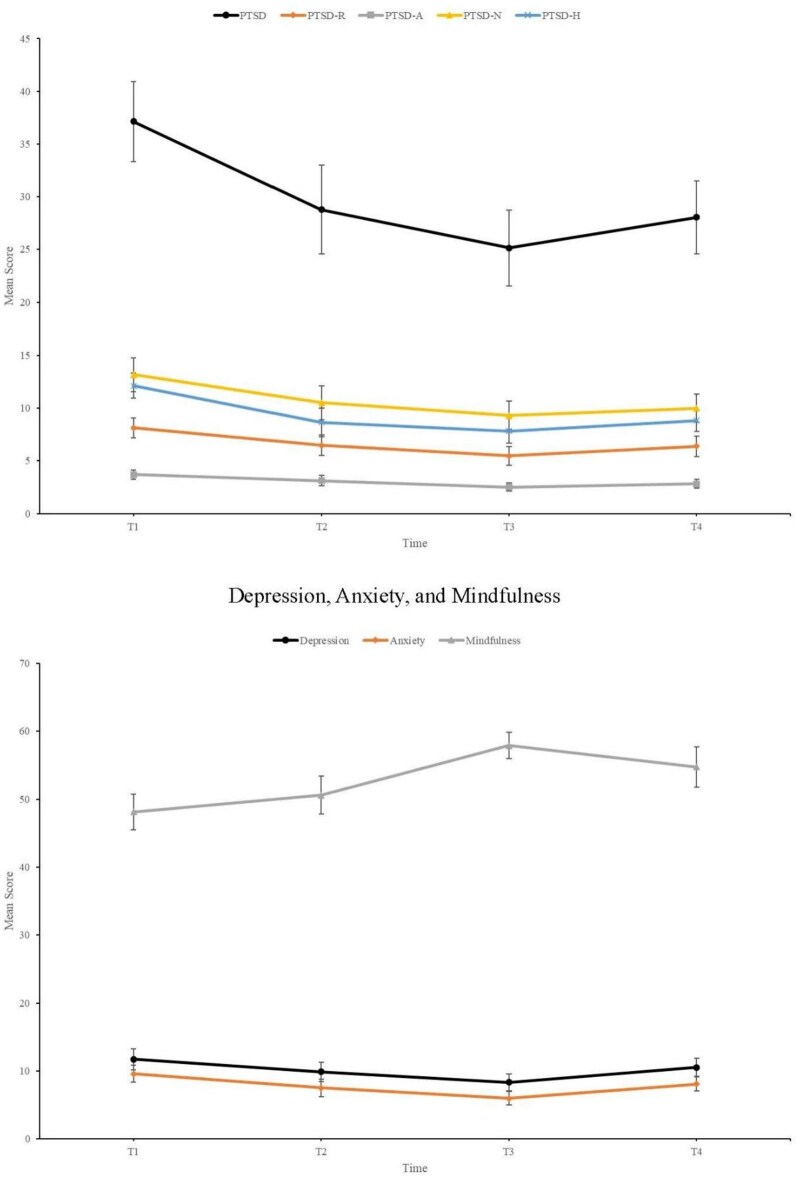
Mean scores of post-traumatic stress disorder (PTSD), and depression, anxiety, and mindfulness scales across 4 timepoints. *Top panel*: Illustration of PTSD and its 4 subscales (PTSD-R, re-experiencing symptoms; PTSD-A, avoidance; PTSD-N, negative alterations in cognition and mood; PTSD-H, hyperarousal) at baseline (T1), mid-treatment 4-weeks from the baseline (T2), post-treatment 8 weeks from the baseline (T3), and follow-up 12 weeks from the baseline (T4). *Bottom panel*: Illustration of depression, anxiety, and mindfulness scores at baseline (T1), mid-treatment 4-weeks from the baseline (T2), post-treatment 8 weeks from the baseline (T3), and follow-up 12 weeks from the baseline (T4).

The mean scores for PTSD, its 4 subscales of symptom clusters (PTSD-R, re-experiencing symptoms; PTSD-A, avoidance; PTSD-N, negative alterations in cognition and mood; PTSD-H, hyperarousal) at T1, T2, T3, and T4 are presented in the top panel of **Figure 1**. The bottom panel of **Figure 1** presents the mean scores for depression, anxiety, and mindfulness across the same time points. A comparison of psychological measures across the 4 time points is presented in **Table 3**.

**Table 3.** Comparison of Psychological Measures Across Timepoints

Variable	M (SD)	M (SD)	*t*	df	*P* (2 sided)	*g* (T1-T2/T3/T4)	95% CI for the effect size
**T1 vs. T2**	**T1**	**T2**					
PTSD	36.73 (17.13)	28.70 (18.70)	3.42	29	0.002	0.61	[0.22, 0.99]
PTSD-R	7.93 (4.49)	6.20 (4.54)	2.79	29	0.009	0.50	[0.12, 0.86]
PTSD-A	3.83 (2.18)	3.17 (2.42)	1.89	29	0.069	0.34	[−0.03, 0.69]
PTSD-N	13.10 (6.87)	10.53 (6.97)	3.04	29	0.005	0.54	[0.16, 0.91]
PTSD-H	11.87 (5.36)	8.80 (6.19)	3.44	29	0.002	0.61	[0.23, 0.99]
Depression	12.03 (6.74)	10.30 (6.33)	2.16	29	0.039	0.38	[0.02, 0.74]
Anxiety	9.93 (5.46)	8.13 (5.79)	1.98	29	0.057	0.35	[−0.01, 0.71]
Mindfulness	49.60 (12.40)	51.30 (12.57)	−0.61	29	0.550	−0.11	[−0.46, 0.24]
**T1 vs. T3**	**T1**	**T3**					
PTSD	37.43 (17.44)	25.61 (16.35)	5.02	27	0.000	0.92	[0.48, 1.35]
PTSD-R	8.14 (4.58)	5.50 (4.13)	4.24	27	0.000	0.78	[0.36, 1.19]
PTSD-A	3.89 (2.18)	2.71 (1.98)	3.18	27	0.004	0.58	[0.19, 0.97]
PTSD-N	13.29 (7.07)	9.36 (6.15)	3.85	27	0.001	0.71	[0.30, 1.11]
PTSD-H	12.11 (5.39)	8.04 (5.34)	5.14	27	0.000	0.95	[0.50, 1.38]
Depression	12.07 (6.95)	8.71 (5.79)	3.55	27	0.001	0.65	[0.25, 1.05]
Anxiety						0.81	[0.39, 1.22]
Mindfulness	49.82 (12.82)	57.96 (8.91)	−2.63	27	0.014	−0.48	[−0.86, −0.10]
**T1 vs. T4**	**T1**	**T4**					
PTSD	37.13 (18.27)	28.04 (16.59)	2.70	22	0.013	0.54	[0.11, 0.97]
PTSD-R	8.13 (4.53)	6.39 (4.64)	1.85	22	0.078	0.37	[−0.04, 0.78]
PTSD-A	3.70 (2.09)	2.83 (1.92)	1.75	22	0.094	0.35	[−0.06, 0.76]
PTSD-N	13.17 (7.67)	10.00 (6.36)	2.19	22	0.039	0.44	[0.02, 0.85]
PTSD-H	12.13 (5.75)	8.83 (4.91)	3.36	22	0.003	0.68	[0.23, 1.11]
Depression	11.74 (7.36)	10.57 (6.25)	0.86	22	0.397	0.17	[−0.23, 0.57]
Anxiety						0.28	[−0.13, 0.68]
Mindfulness	48.17 (12.62)	54.83 (14.28)	−2.03	22	0.055	−0.41	[−0.82, 0.01]

Comparison of PTSD, depression, and anxiety scales at baseline (T1) versus mid-treatment 4-weeks from the baseline (T2), versus post-treatment 8 weeks from the baseline (T3), and versus follow-up 12 weeks from the baseline (T4).

*N*_T1-T2_ = 30; *N*_T3_ = 28; *N*_T4_ = 23.

Abbreviations: PTSD, post-traumatic stress disorder; PTSD-A, post-traumatic stress disorder avoidance; PTSD-H, post-traumatic stress disorder hyperarousal; PTSD-N, post-traumatic stress disorder negative alterations in cognition and mood; PTSD-R, post-traumatic stress disorder re-experiencing symptoms.

#### Post-traumatic stress disorder

Over time, significant changes in PTSD symptoms were observed (*χ*^2^(3) = 15.74, *P* = .001). A significant large effect was observed from baseline to in-session (*r* = 0.69, *Z *=* −*3.22, *P* = .001), and at post-intervention (*r* = 0.78, *Z *=* −*3.67, *P* < .001). Although there was a slight increase at follow-up, the effect size remained large, indicating a significant decrease from baseline (*r* = 0.55, *Z *=* −*2.63, *P* = .008).

#### PTSD symptom clusters

The post-traumatic stress disorder subscales of PTSD symptom clusters showed similar trends. Re-experiencing symptoms significantly changed over time (*χ*^2^(3) = 16.28, *P* < .001), with a large effect observed from baseline to in-session (*r* = 0.64, *Z *=* −*2.94, *P* = .003), and at post-intervention (*r* = 0.73, *Z *=* −*3.43, *P* < .001). Although there was a slight increase at follow-up, the effect size remained moderate, but did not reach significance (*r* = 0.42, *Z *=* −*1.80, *P* = .07).

Avoidance symptoms also showed significant changes (*χ*^2^(3) = 9.02, *P* = .03). A large effect size was observed from baseline to in-session (*r* = 0.58, *Z *=* −*2.23, *P* = .02), but this did not reach the Bonferroni-adjusted significance level of 0.017. A large effect was observed from baseline to post-intervention (*r* = 0.68, *Z *=* −*2.71, *P* = .007). Although a slight increase was noted at follow-up, the effect size remained moderate (*r* = 0.34, *Z *=* −*1.40, *P* = .16), however, significance was not reached.

Negative alterations in cognition and mood followed a similar pattern (*χ*^2^(3) = 13.00, *P* = .005). Scores decreased from baseline to in-session (*r* = 0.63, *Z *=* −*2.89, *P* = .004), and post-intervention (*r* = 0.70, *Z *=* −*3.22, *P* = .001) both with large effects. Scores remained below baseline at follow-up, with a moderate effect (*r* = 0.39, *Z *=* −*1.78, *P* = .08), though this change was not statistically significant.

Hyperarousal symptoms showed significant changes as well (*χ*^2^(3) = 21.46, *P* < .001). Scores decreased from baseline to in-session (*r* = 0.63, *Z *=* −*2.95, *P* = .003) and to post-intervention (*r* = 0.84, *Z *=* −*3.85, *P* < .001), both with a large effect. Although a slight increase was observed at follow-up, scores remained below baseline with a large effect (*r* = 0.64, *Z *=* −*2.93, *P* = .003).

#### Depression

Depression scores also changed significantly over time (*χ*^2^(3) = 9.93, *P* = .02). Baseline to in-session scores decreased with a moderate effect (*r* = 0.39, *Z *=* −*1.78, *P* = .074), though this did not reach statistical significance. A large effect was observed from baseline to post-intervention (*r* = 0.67, *Z *=* −*3.09, *P* = .002), which was statistically significant. Although a slight increase was observed at follow-up, scores remained below baseline (*r* = 0.23, *Z *=* −*1.06, *P* = .288), but this change was not significant.

#### Anxiety

Anxiety scores showed significant changes over time (*χ*^2^(3) = 9.93, *P* = .03). A moderate effect was observed from baseline to in-session (*r* = 0.36, *Z *=* −*1.64, *P* = .102), though this did not reach statistical significance. In contrast, the decrease from baseline to post-intervention was associated with a large effect size (*r* = 0.75, *Z *=* −*3.44, *P* < .001). Scores remained below baseline at follow-up (*r* = 0.27, *Z *=* −*1.13, *P* = .257), however, the effect was not significant.

#### Mindfulness

Mindfulness scores showed no statistically significant changes over time (*χ*^2^(3) = 5.83, *P* = .12). Although the overall Friedman test did not reach statistical significance, exploratory post-hoc Wilcoxon signed-rank tests were conducted to examine potential patterns of change; these results should be interpreted with caution. A small effect was observed from baseline to in-­session (*r* = 0.07, *Z *=* −*0.33, *P* = .742), however, significance was not reached. A moderate effect was found from baseline to post-intervention (*r* = 0.55, *Z *=* −*2.63, *P* = .008), which was statistically significant. Although follow-up scores remained moderately elevated compared to baseline (*r* = 0.35, *Z *=* −*1.69, *P* = .091), this change did not reach significance.

## Discussion

This study examined the effectiveness of a tailored TI-MBSR intervention for ADF veterans, focusing on its impact on PTSD, depression, anxiety, and mindfulness. Using a repeated-­measures design, symptom changes were assessed at 4 time points: pre-intervention (T1), mid-intervention (T2), post-intervention (T3), and 2-month follow-up (T4). The study hypothesized significant reductions in PTSD symptoms across its 4 subdomains—re-experiencing, avoidance, negative alterations in cognition and mood, and hyperarousal—as well as decreases in depression and anxiety symptoms, alongside increases in mindfulness levels over time.

### PTSD Symptoms

The findings revealed significant improvements in PTSD symptoms, including the sub-domains of re-experiencing, avoidance, negative alterations in cognition and mood, and hyperarousal, with large effect sizes observed post-intervention. Although some symptom resurgence was noted at follow-up, levels remained significantly lower than baseline, suggesting sustained benefits. These outcomes align with existing PTSD research supporting MBSR as an effective, non-trauma-focused intervention that minimizes the risk of emotional overwhelm and re-traumatization—common barriers in traditional trauma therapies.^16^

A notable advantage of TI-MBSR is its non-pharmacological and non-invasive nature. Although psychotropic medications are often used to manage PTSD symptoms, they can come with side effects, dependency risks, or stigma, particularly in military settings.^4^^,^^18^ Trauma-Informed Mindfulness Based Stress Reduction offers a complementary or alternative approach, free from these drawbacks, empowering veterans to manage their symptoms independently.^16^

### Depression and Anxiety Symptoms

The findings revealed significant reductions in depression and anxiety symptoms across the TI-MBSR intervention, with marked improvements observed from baseline to mid-intervention and further gains by post-intervention. These results align with existing evidence supporting MBSR as an effective intervention for addressing the core psychological mechanisms underpinning depression and anxiety, including rumination, emotional dysregulation, and catastrophic thinking.^12^

A central strength of TI-MBSR lies in its ability to address rumination, a cognitive pattern characterized by persistent dwelling on past negative thoughts and emotions,^30^ which is strongly associated with depression.^31^ Mindfulness meditation enhances focused attention on the present moment, which may interrupt ruminative thought patterns by redirecting attention away from the past.^30^ This subtle shift created space for self-compassion, counteracting self-blame and guilt—­emotions frequently intensified by military cultural norms emphasizing resilience and self-reliance.^1^^,^^18^ Equally important is the capacity for TI-MBSR to improve emotional regulation, a shared vulnerability in PTSD, depression, and anxiety.^32^ Emotional dysregulation often manifests as heightened emotional reactivity, difficulty returning to baseline after stress, or impulsive reactions. Mindfulness meditation can enable veterans to pause, observe their emotional states, and respond thoughtfully rather than react impulsively.^13^

However, although mean depression and anxiety scores remained lower than pre-intervention levels at the 2-month follow-up, the differences were not statistically significant, suggesting that the benefits were not sustained after 2 months. The diminishment of the effect over time may be because of participants discontinuing regular mindfulness meditation after the intervention. Without ongoing engagement, the benefits of TI-MBSR, such as reducing the symptoms of anxiety and depression, may not be sustained. This finding suggests that continued practice is critical for maintaining gains and highlights the need for follow-up support or strategies to encourage long-term adherence.

### Mindfulness State

Mindfulness scores, as a measure of momentary mindful awareness or state mindfulness, did not significantly change across the 4 time points, suggesting that the TI-MBSR intervention may not have produced consistent, measurable shifts in present-moment awareness during the study period. Several factors may explain this finding. First, mindfulness as a state is inherently variable and may require extended and sustained practice to stabilize and be reliably captured in self-report measures. Second, individual differences in engagement with mindfulness practices, prior experience, or psychological readiness may have influenced the capacity to access or report state mindfulness consistently. Third, the measurement itself may not fully capture early or subtle shifts, especially in populations unfamiliar with the language of mindfulness.

Despite the lack of statistically significant change in state mindfulness, the practice of mindfulness—as delivered through the TI-MBSR intervention—appeared to meaningfully support improvements in PTSD, depression, and anxiety symptoms. This suggests that even in the absence of significant self-­reported changes in mindful awareness, participants may still have benefited from the structured engagement with mindfulness-based strategies. Exploratory comparisons indicated a moderate increase from baseline to post-intervention, which, although not conclusive, aligns with the theoretical mechanisms by which mindfulness practices are thought to enhance emotional regulation and reduce psychological distress.^13^^,^^14^

### Limitations

Several limitations exist in the current study. First, the study’s convenient sampling increases the risk of selection bias.^32^ Participants may have been more motivated or predisposed to engage with mindfulness, potentially overestimating the effectiveness of the MBSR program. Moreover, the use of a single-arm design precluded direct comparisons with a control group. However, when comparing our findings with those of Li et al. (2024) in their meta-analysis,^13^ our results for depression and PTSD at T1 (baseline) and T3 (post-treatment) were generally comparable to the pooled estimates, which demonstrated significant treatment effects for these conditions. Our study observed a decline in effect sizes at T4 similar to Li et al. (2024),^13^ with depression and mindfulness showing non-significant differences between T1 and T4. Our study, however, showed a significant improvement from T1 to T3 in PTSD symptoms. Future research should use RCT designs to strengthen causal inferences and incorporate waitlist control or active comparator groups for clearer assessments.^33^

Second, the small sample size limits generalizability. Although effects were statistically significant, smaller samples reduce statistical power, increasing the risk of Type II errors and susceptibility to outliers.^34^ Moreover, while our study did not find a significant effect for mindfulness, this may be attributed to the limited statistical power resulting from our small sample size. Larger, more diverse samples and multi-site studies across different regions are needed to improve external validity and reduce demographic and geographic biases.^35^

Third, the limited representation of female veterans in this study highlights a significant gap. Women veterans face unique mental health challenges, formed only a minority of the current study’s participants. Their underrepresentation means our findings may not fully capture their experiences with TI-MBSR. Future research should employ targeted recruitment strategies to enhance female participation in veteran mental health studies.

Fourth, symptom resurgence at follow-up suggests that while TI-MBSR provides significant short-term benefits, continued support is essential for maintaining gains.^14^ Regular mindfulness meditation and reinforcement are crucial to the effectiveness of TI-MBSR.^36^ Incorporating booster sessions, refresher workshops, or peer-led mindfulness groups could help sustain long-term outcomes. Additionally, mobile applications, guided recordings, and online mindfulness communities could provide scalable options for continued engagement.^37^

Furthermore, this study is based on quantitative findings, which, while valuable for assessing symptom changes, do not capture the lived experiences of veterans undergoing TI-MBSR. A qualitative approach would provide deeper insight into how veterans perceive and engage with mindfulness meditation, including facilitators and barriers to the effectiveness of TI-MBSR.^1^ Future research should integrate qualitative methodologies, such as interviews or focus groups, to enrich the understanding of veterans’ experiences and the nuanced impacts of MBSR.

Beyond these limitations, broader structural and cultural factors may have influenced outcomes.^4^^,^^15^ Military culture, emphasizing stoicism, resilience, and self-reliance, may conflict with the openness encouraged in MBSR.^18^ Future research should explore how these pre-existing attitudes affect engagement and outcomes in mindfulness programs.

### Clinical Implications

The findings from this study hold significant clinical implications for addressing the mental health needs of veterans, a population that often faces unique barriers to accessing and benefiting from traditional mental health interventions. Trauma-Informed Mindfulness Based Stress Reduction emerges as a viable, non-pharmacological intervention for managing symptoms of PTSD, depression, and anxiety, offering a complementary or alternative approach to conventional treatments such as trauma-focused therapies or psychotropic medications. This distinction is particularly important in military culture, where there is often stigma surrounding the use of medications and a reluctance to engage in therapies that require direct confrontation with traumatic memories.^4^^,^^18^

#### Trauma-Informed approach

Trauma-Informed Mindfulness Based Stress Reduction presents a clinical advantage and offers significant value for engaging veterans who may be hesitant about trauma-focused therapies within military and community mental health services. Unlike therapies that require direct confrontation with traumatic memories, TI-MBSR focuses on building emotional regulation and resilience through mindfulness techniques,^14^ and emphasizes establishing safety, trustworthiness empowerment, and choice.^22^ This indirect approach allows veterans to address trauma-related symptoms gradually, minimizing the risk of emotional overwhelm or treatment dropout. For clinicians, this means TI-MBSR can serve as an effective entry point into therapeutic engagement for individuals who might otherwise avoid seeking help. Furthermore, the non-judgmental, accepting framework of mindfulness empowers veterans to approach their symptoms with curiosity rather than fear, reframing their relationship with distressing emotions and physical sensations.^13^

#### Integration into existing mental health services

Clinicians, policy makers, and organizations providing mental health services to veterans should consider integrating TI-MBSR into their treatment portfolios, either as a standalone intervention or as an adjunct to existing therapies. TI-MBSR could be integrated into military transition services, rehabilitation and recovery programs, and alongside traditional psychotherapies which support veterans. It could also serve as a preparatory or stabilizing intervention for veterans who are considering, but not yet ready for, trauma-focused therapies.^38^ By building foundational skills in self-awareness, emotional regulation, and non-reactivity, TI-MBSR can increase veterans’ readiness to engage in more intensive psychological treatments if needed.^21^^,^^22^

#### Sustainability and long-term engagement

One of the critical insights from this study is the observed slight resurgence of symptoms during the follow-up period, highlighting the importance of sustaining engagement with mindfulness meditation over time. Clinicians and service providers should consider implementing booster sessions, refresher workshops, or ongoing group meetings to maintain the therapeutic gains achieved during the initial intervention.^39^ These follow-up engagements could be delivered in flexible formats, such as in-person sessions, virtual meetings, or app-based guided meditations, to ensure accessibility and convenience for veterans who may have scheduling or geographic constraints. Additionally, providing veterans with home practice resources, such as guided meditation recordings and mindfulness journals, can encourage independent practice and reinforce long-term habit formation.^14^

#### Training and education for clinicians

To optimize the implementation of TI-MBSR, it is essential for clinicians and facilitators to receive specialized training in MBIs and trauma-sensitive frameworks. Veterans may present with complex trauma histories and unique cultural considerations that require sensitive facilitation. Clinicians who are trained in both mindfulness techniques and trauma-informed care will be better equipped to create a supportive environment, address resistance or skepticism, and navigate the emotional nuances that may arise during TI-MBSR sessions.

#### Cost-effectiveness and scalability

From a broader health services perspective, TI-MBSR represents a cost-effective intervention that can be delivered in group formats, thereby reaching more participants with fewer resources compared to one-on-one therapy sessions. The scalability of TI-MBSR also makes it an appealing option for veteran mental health services, particularly in resource-limited settings. Online adaptations of MBSR have also shown promise and could further increase accessibility for veterans living in remote or underserved regions.^37^

## CONCLUSION

The findings of this study underscore the significant clinical value of TI-MBSR for addressing PTSD, depression, and anxiety in veterans. The results demonstrated significant reductions in symptoms of PTSD, including improvements across the subdomains of re-experiencing, avoidance, negative alterations in cognition and mood, and hyperarousal. Additionally, depression and anxiety symptoms showed marked decreases during the intervention period; however, these reductions diminished at follow-up. Mindfulness state scores did not show statistically significant increases across time points; however, exploratory trends suggested a moderate post-intervention improvement, which may reflect early shifts in present-moment awareness and emotional regulation that potentially contributed to the observed reductions in PTSD, depression, and anxiety.

It is important to note that the findings should be interpreted with caution because of the small sample size in our study. This limitation may have introduced bias, which potentially influences the outcomes and limiting the generalizability of the results.

Trauma-Informed Mindfulness Based Stress Reduction structured yet adaptable format, trauma-informed approach, and emphasis on emotional self-regulation makes it well-suited to the unique psychological and cultural needs of military personnel. Mental health services should focus on integrating TI-MBSR into broader veteran treatment frameworks and pathways, ensuring sustained engagement through follow-up strategies, and addressing gaps in representation and facilitator training. By doing so, TI-MBSR can serve as a cornerstone intervention for improving mental health outcomes and overall well-being among veterans.

## Supplementary Material

usaf393_Supplementary_Data

## Data Availability

The data underlying this article are available in Research Dats JCU, at https://doi.org/10.25903/t6tz-n138

## INSTITUTIONAL CLEARANCE

Institutional clearance does not apply.
