# Supplementary material for: Mindfulness Intervention for Navigating and Decreasing Stress (MINDS-V): Effectiveness of a Tailored Trauma-Informed Mental Health Intervention for Australian Veterans
Source: Mil Med. 2025 Aug 6;191(1-2):e319–29. doi: 10.1093/milmed/usaf393 (PMC12828227; doi:10.1093/milmed/usaf393)

**Supplementary Materials**

**Table S1**

*Tailored intervention: summary of adaptions for Australian military veterans*

| Tailored Adaptions | | Example |
| --- | --- | --- |
| *Military / Veteran Specific* | |  |
|  | Acknowledging military / veteran mindset, values, and experiences |  |
|  | Non-clinical environment including community gym / university campus |  |
|  | Research team knowledgeable about and familiar with military / veteran culture, values an experiences |  |
|  | Instructor familiarity with military / veteran culture and context |  |
|  | Adjustments to mindfulness exercises to accommodate physical limitations or injuries common among veterans | Seated / standing / lying down mediation options  Provision of modified mindful movement practices and poses  Keep eyes open during meditations  Mindful movement / walking instead of stillness |
|  | Language modifications to reflect military culture | Recognising and acknowledging the military service of participants |
|  |  | Using terminology relevant to military populations e.g. “deployment”, “service members”, “civvy” |
| *Trauma-Informed Approach: prioritises safety, trust, and empowerment ensuring that all interactions are sensitive to the impact of trauma* | |  |
|  | Provision of a secure and confidential research environment |  |
|  | Orientation session with MBSR instructor |  |
|  | Language modifications | Avoiding medical or psychological jargon where possible  Using trauma sensitive language such as military experience |
|  | Adapting MBSR terminology to encourage engagement and accessibility | Referring to yoga as mindful movement or mindful stretching |
|  | Group-based peer support to foster a sense of shared understanding, mutual support, and trust | Participation limited to veterans to cultivate shared group understanding |
|  |  |  |
|  | Emphasis on personal safety and control within the class situation, and encouraging participants to take an active role in their participation and supporting choice | Use of language that empowers veterans and respects their autonomy, such as "You have the choice to..." or "It's up to you if...” |
|  | Support veterans in advocating for their own mental health needs and preferences within the research process | Provide resources that support veterans in their advocacy efforts, such as information on mental health services, and connections to veteran support organisations  Encourage participants to share their mindfulness / research experiences with family / friends to support their own mental health needs and progress |
|  | Acknowledge challenges, effort and positive experiences of participants throughout the research process |  |

**Table S2**

*Spearman’s Correlations and Means (Standard Deviations) of PTSD, Depression, and Anxiety Between T1, T2, T3, and T4*

*Note. N_T1_ = 41; N_T2_ = 30; N_T3_ = 28; N_T4_ = 23*

**p < 0.05; **p < 0.01*

*PTSD-R, re-experiencing symptoms; PTSD-A, avoidance; PTSD-N, negative alterations in cognition and mood; PTSD-H, hyperarousal.*

**Table S3**

*Shapiro-Wilk Test of Normality*

|  | Statistic | df | Sig. |
| --- | --- | --- | --- |
| T1 PTSD | 0.980 | 23 | 0.907 |
| T1 PTSD-R | 0.939 | 23 | 0.175 |
| T1 PTSD-A | 0.930 | 23 | 0.112 |
| T1 PTSD-N | 0.959 | 23 | 0.448 |
| T1 PTSD-H | 0.968 | 23 | 0.646 |
| T1 Depression | 0.937 | 23 | 0.158 |
| T1 Anxiety | 0.949 | 23 | 0.279 |
| T1 Mindfulness | 0.977 | 23 | 0.847 |
| T2 PTSD | 0.908 | 23 | 0.038 |
| T2 PTSD-R | 0.939 | 23 | 0.172 |
| T2 PTSD-A | 0.935 | 23 | 0.138 |
| T2 PTSD-N | 0.911 | 23 | 0.043 |
| T2 PTSD-H | 0.898 | 23 | 0.023 |
| T2 Depression | 0.907 | 23 | 0.035 |
| T2 Anxiety | 0.867 | 23 | 0.006 |
| T2 Mindfulness | 0.967 | 23 | 0.624 |
| T3 PTSD | 0.915 | 23 | 0.051 |
| T3 PTSD-R | 0.929 | 23 | 0.103 |
| T3 PTSD-A | 0.908 | 23 | 0.038 |
| T3 PTSD-N | 0.941 | 23 | 0.186 |
| T3 PTSD-H | 0.914 | 23 | 0.049 |
| T3 Depression | 0.938 | 23 | 0.163 |
| T3 Anxiety | 0.919 | 23 | 0.064 |
| T3 Mindfulness | 0.975 | 23 | 0.807 |
| T4 PTSD | 0.932 | 23 | 0.124 |
| T4 PTSD-R | 0.930 | 23 | 0.111 |
| T4 PTSD-A | 0.920 | 23 | 0.065 |
| T4 PTSD-N | 0.941 | 23 | 0.188 |
| T4 PTSD-H | 0.956 | 23 | 0.379 |
| T4 Depression | 0.966 | 23 | 0.584 |
| T4 Anxiety | 0.918 | 23 | 0.059 |
| T4 Mindfulness | 0.967 | 23 | 0.609 |

*Note.* Measurements at Baseline (T1), mid-treatment four-weeks from the baseline (T2), post-treatment eight weeks from the baseline (T3), follow-up 12 weeks from the baseline (T4).

PTSD-R, re-experiencing symptoms; PTSD-A, avoidance; PTSD-N, negative alterations in cognition and mood; PTSD-H, hyperarousal.

**Figure S1**

*T1 PTSD Q-Q Plot*


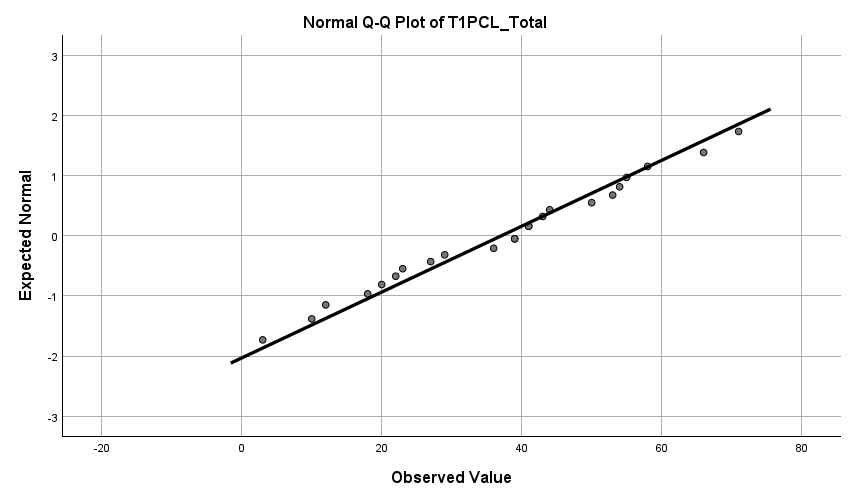


*T1 PTSD-R Q-Q Plot*


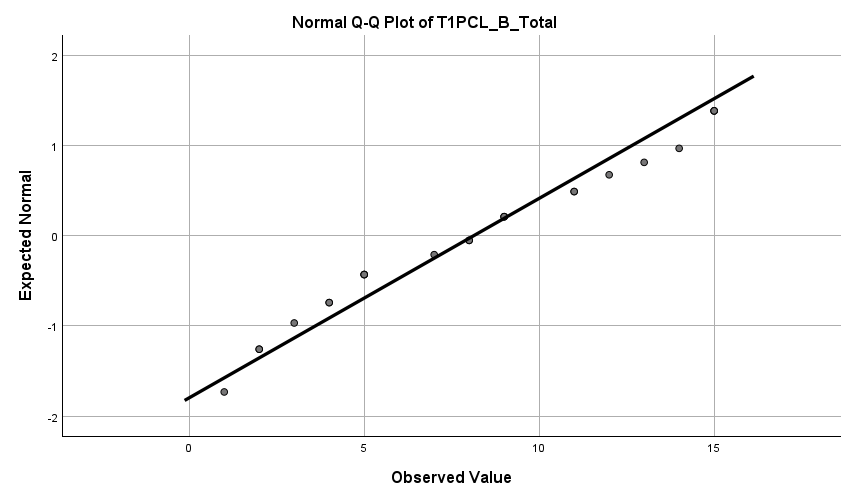


*T1 PTSD-A Q-Q Plot*

*
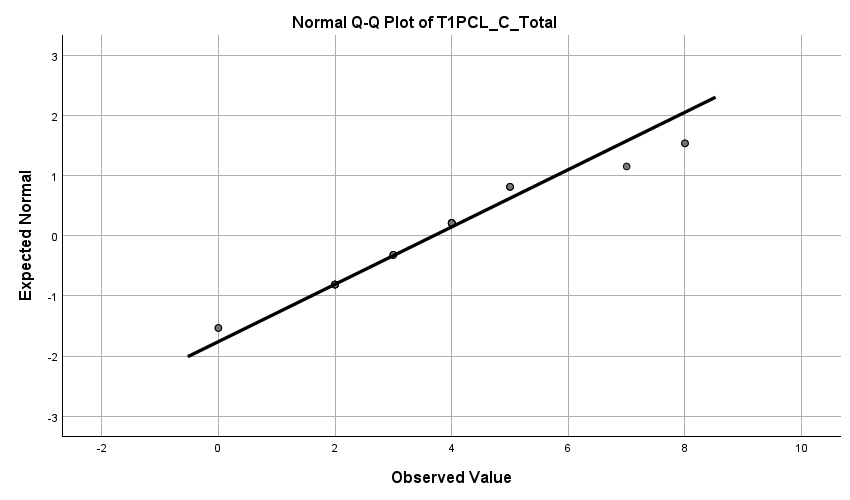
*

*T1 PTSD-N Q-Q Plot*

*
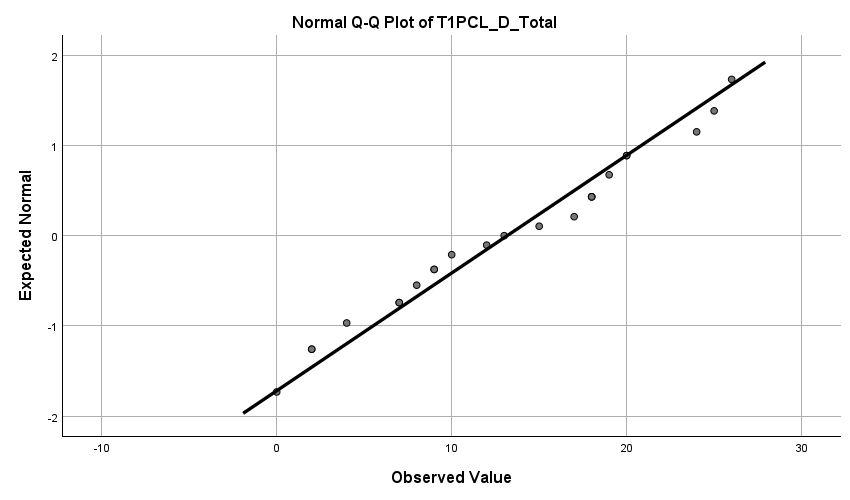
*

*T1 PTSD-H Q-Q Plot*

*
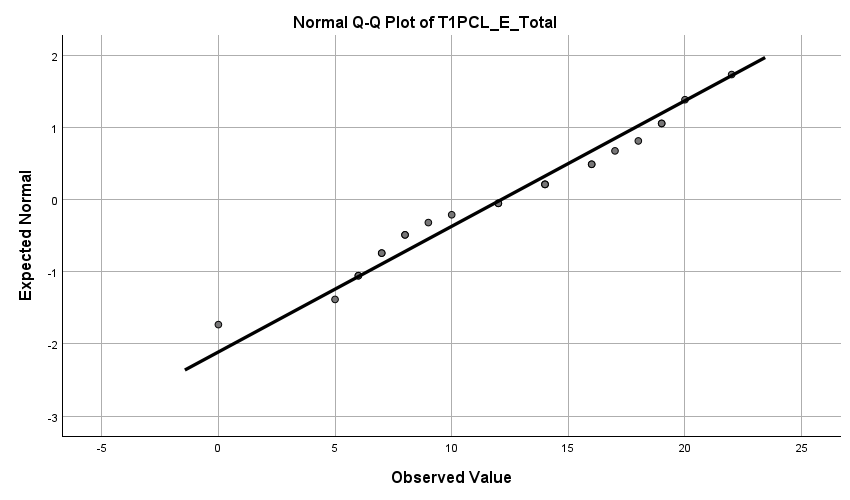
*

*T1 Depression Q-Q Plot*

*
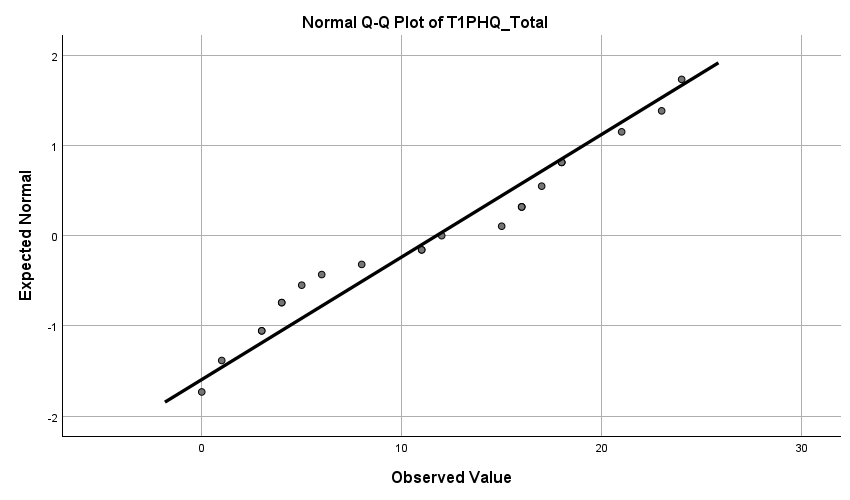
*

*T1 Anxiety Q-Q Plot*

*
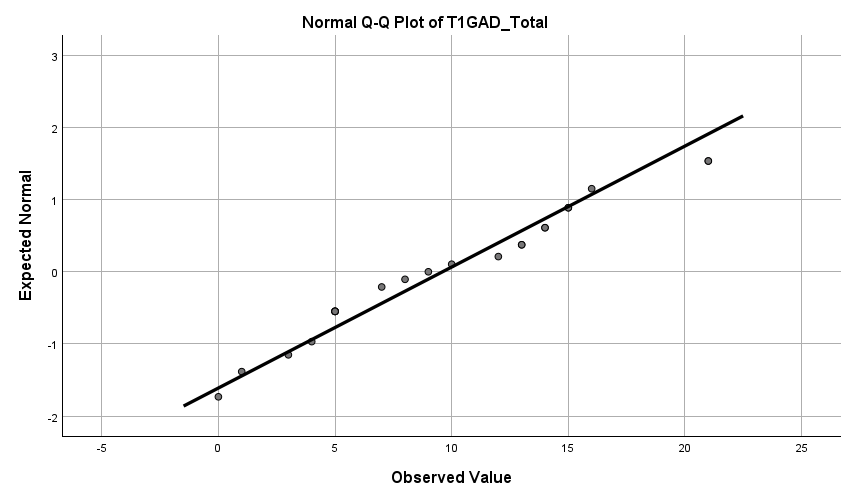
*

*T1 Mindfulness Q-Q Plot*

*
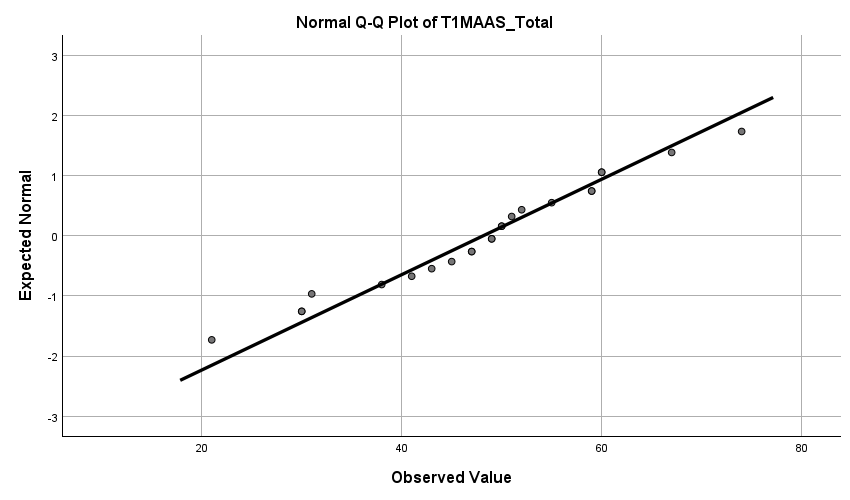
*

*T2 PTSD Q-Q Plot*

*
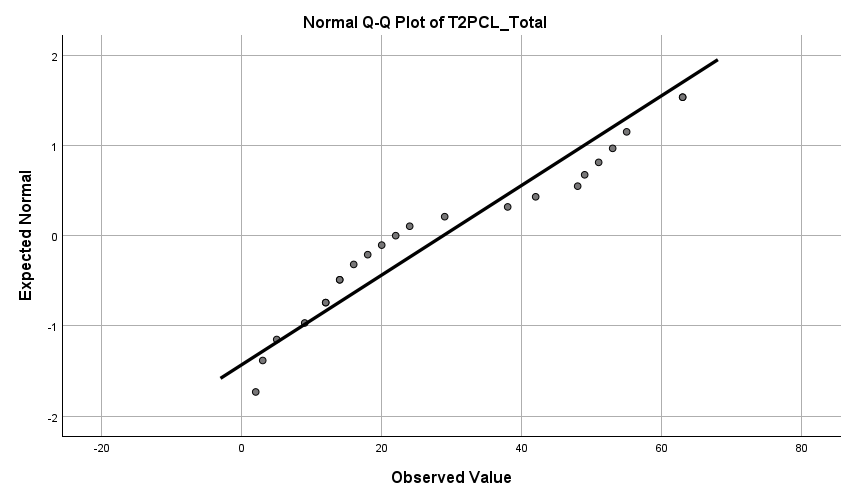
*

*T2 PTSD-R Q-Q Plot*

*
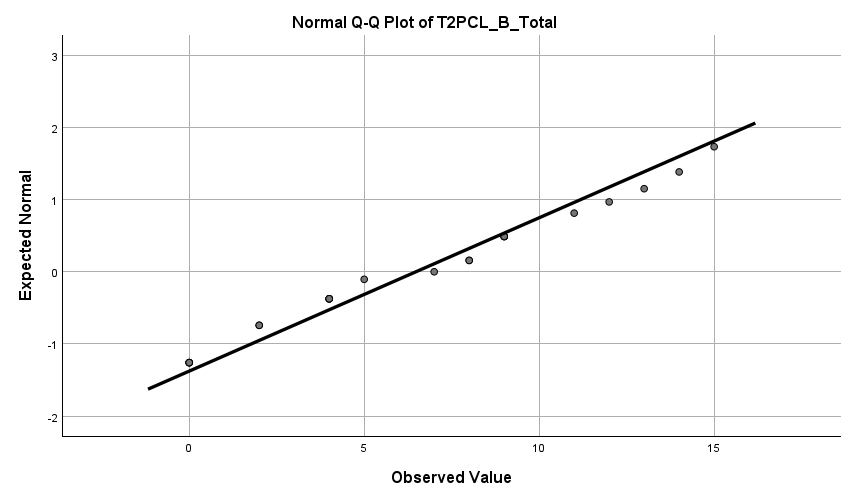
*

*T2 PTSD-A Q-Q Plot*

*
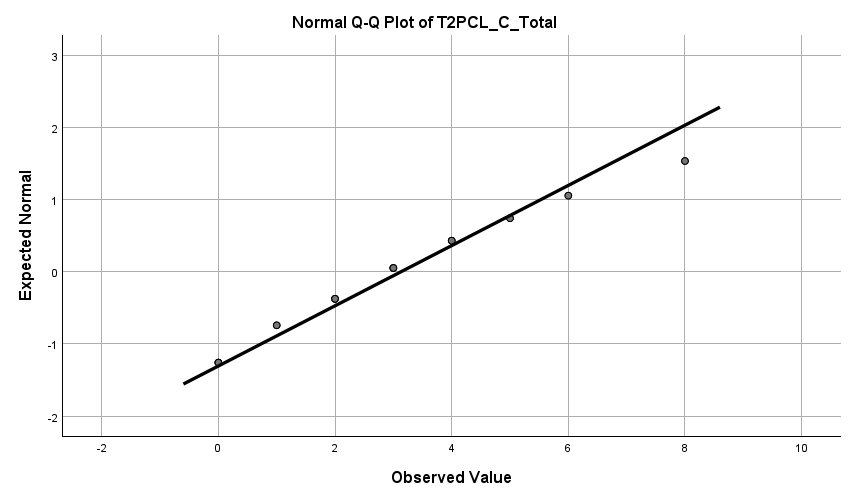
*

*T2 PTSD-N Q-Q Plot*

*
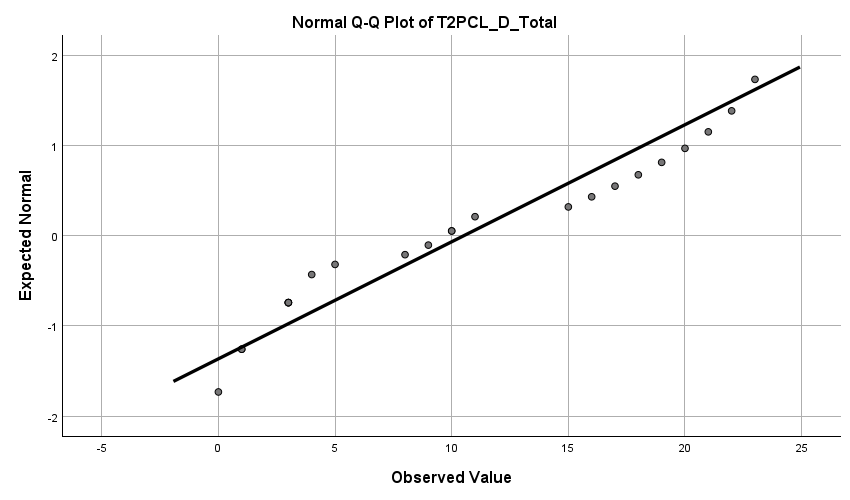
*

*T2 PTSD-H Q-Q Plot*

*
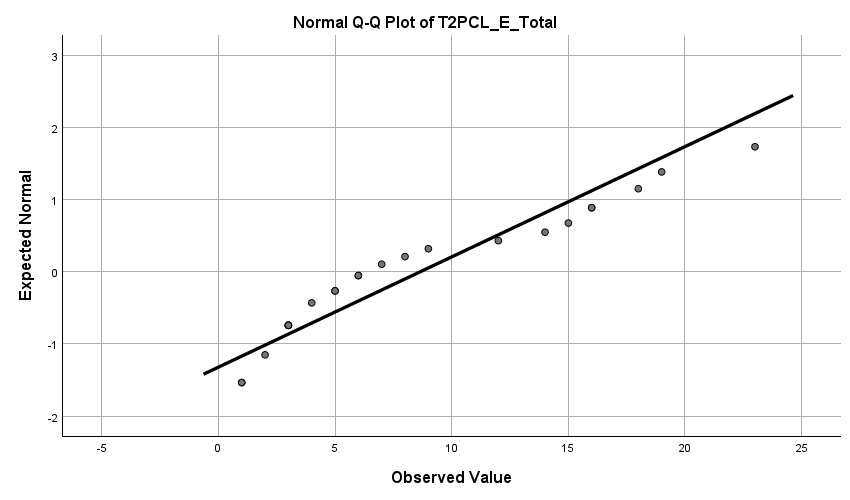
*

*T2 Depression Q-Q Plot*

*
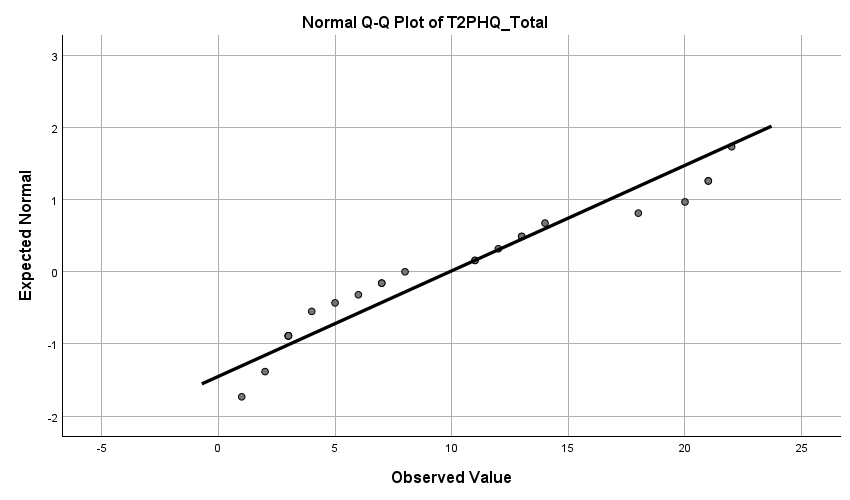
*

*T2 Anxiety Q-Q Plot*

*
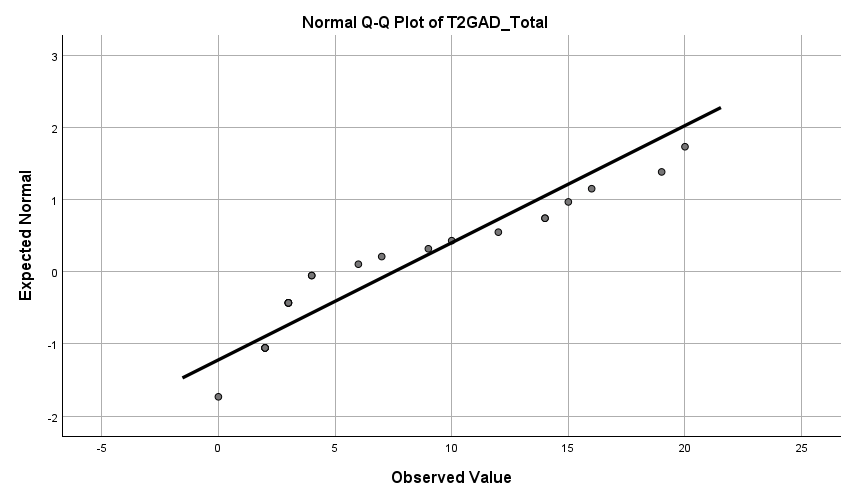
*

*T2 Mindfulness Q-Q Plot*

*
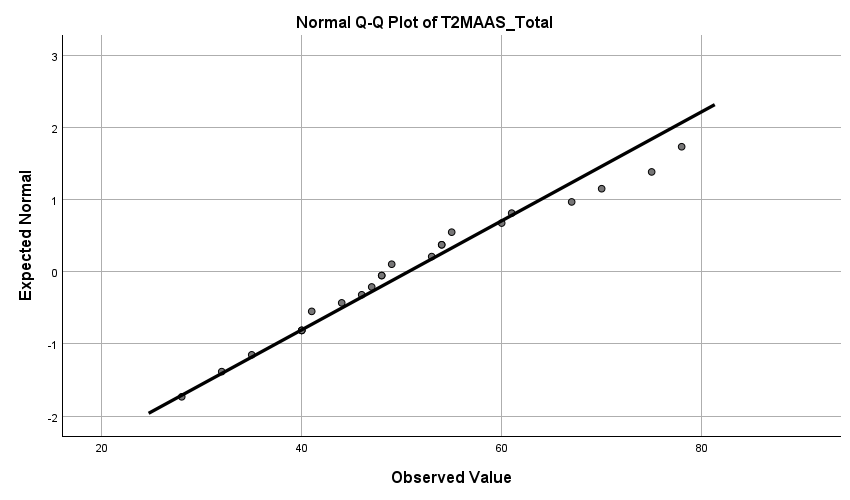
*

*T3 PTSD Q-Q Plot*

*
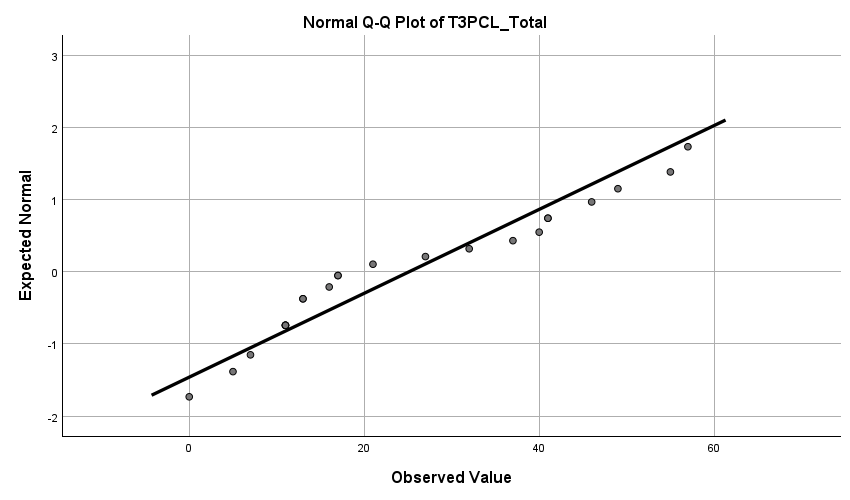
*

*T3 PTSD-R Q-Q Plot*

*
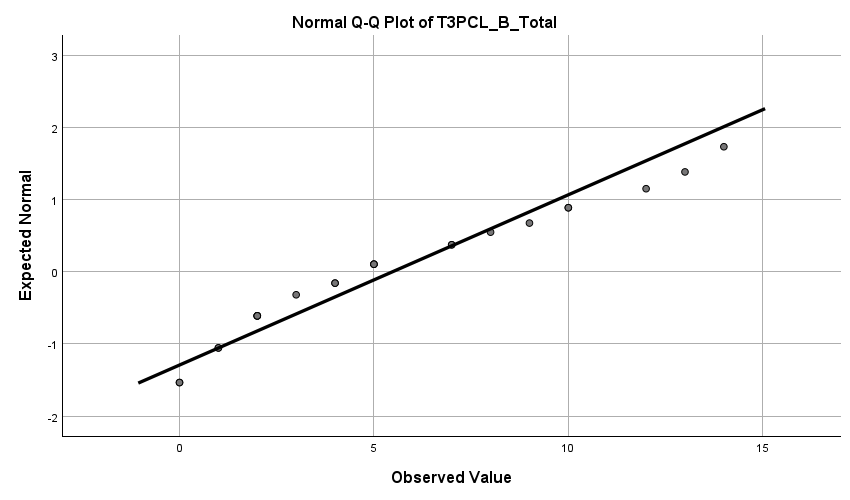
*

*T3 PTSD-A Q-Q Plot*

*
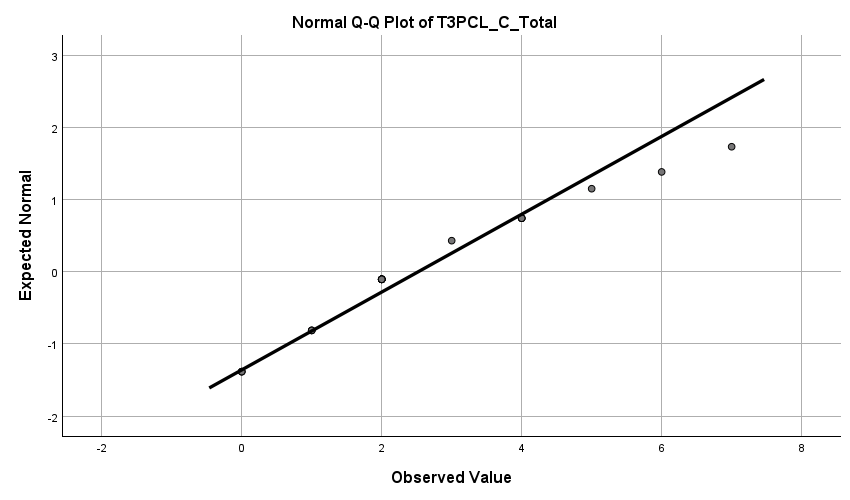
*

*T3 PTSD-N Q-Q Plot*

*
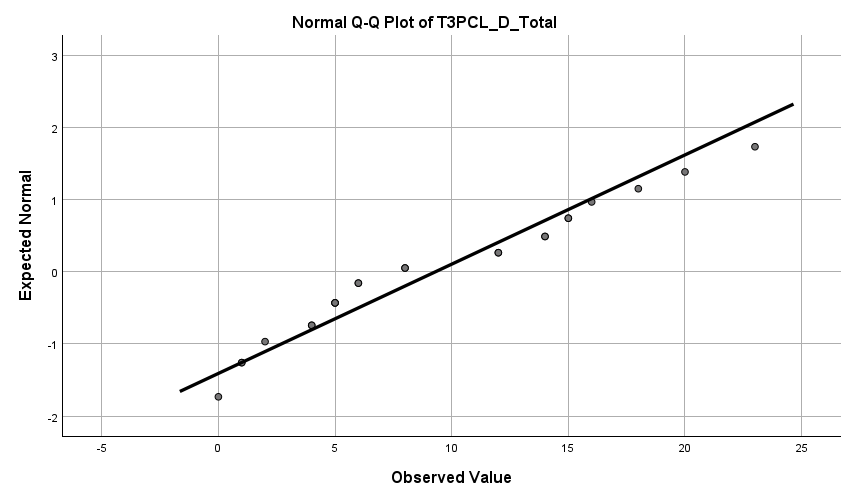
*

*T3 PTSD-H Q-Q Plot*

*
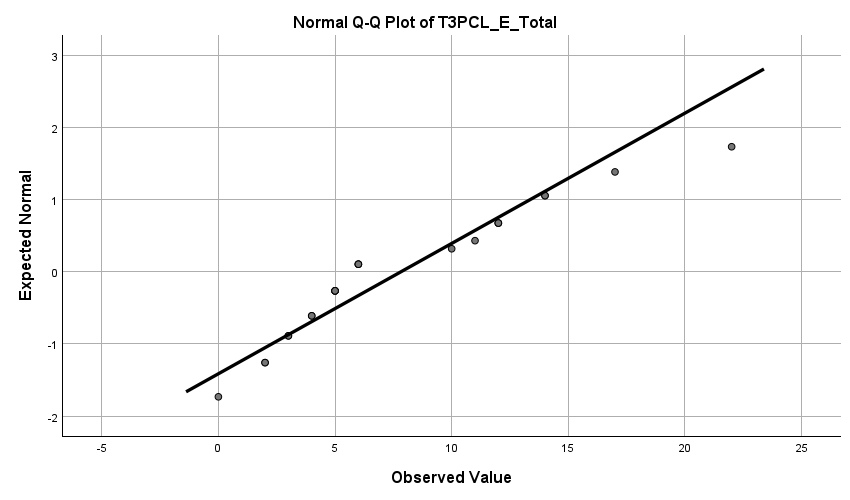
*

*T3 Depression Q-Q Plot*

*
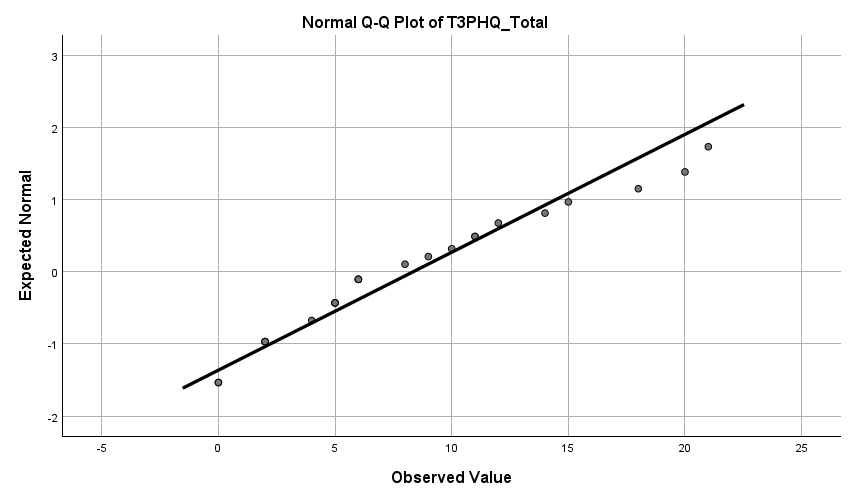
*

*T3 Anxiety Q-Q Plot*

*
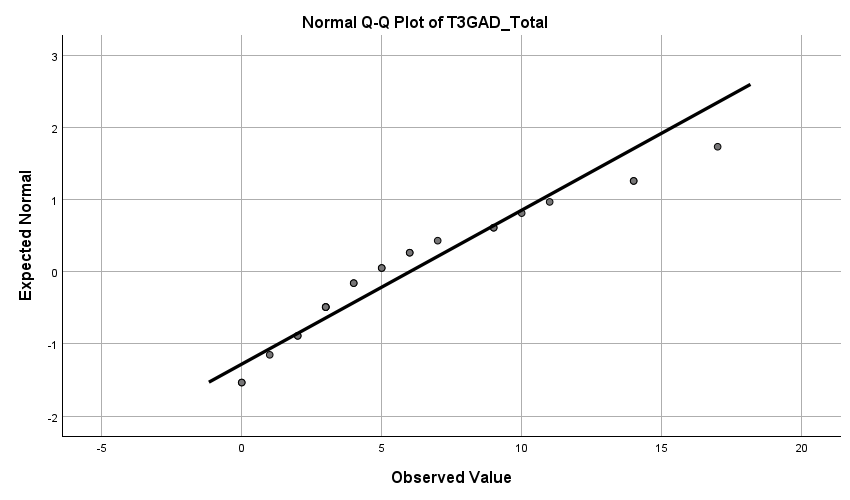
*

*T3 Mindfulness Q-Q Plot*

*
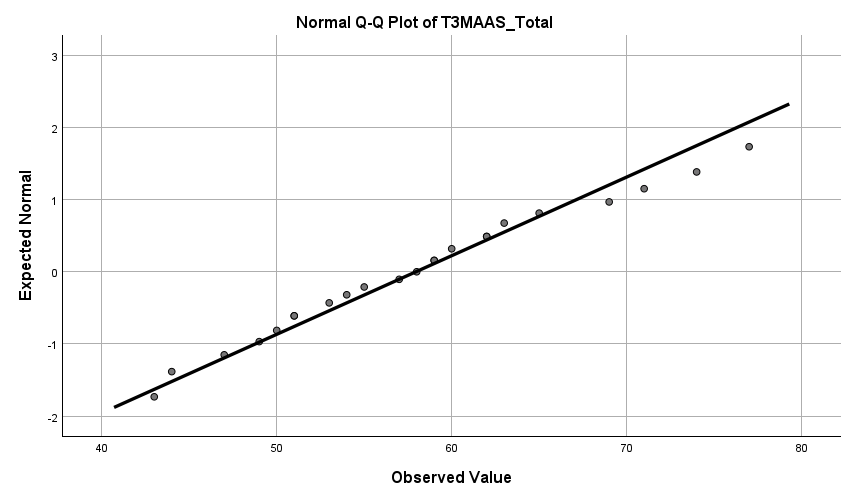
*

*T4 PTSD Q-Q Plot*

*
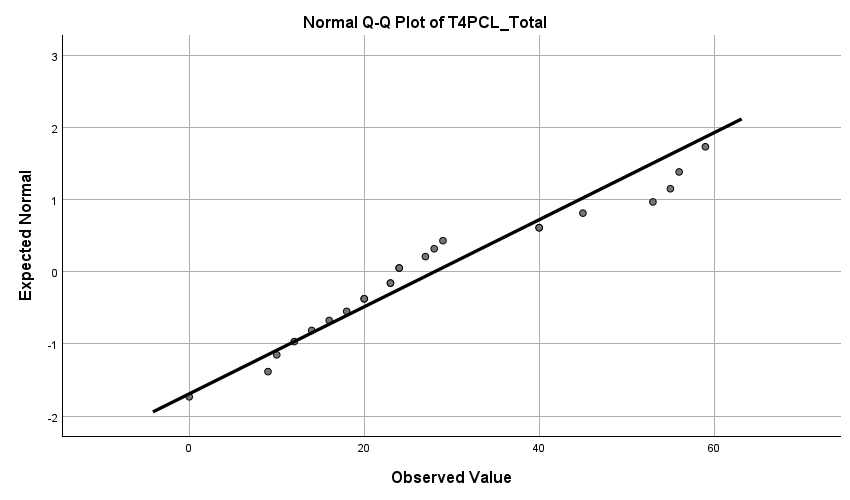
*

*T4 PTSD-R Q-Q Plot*

*
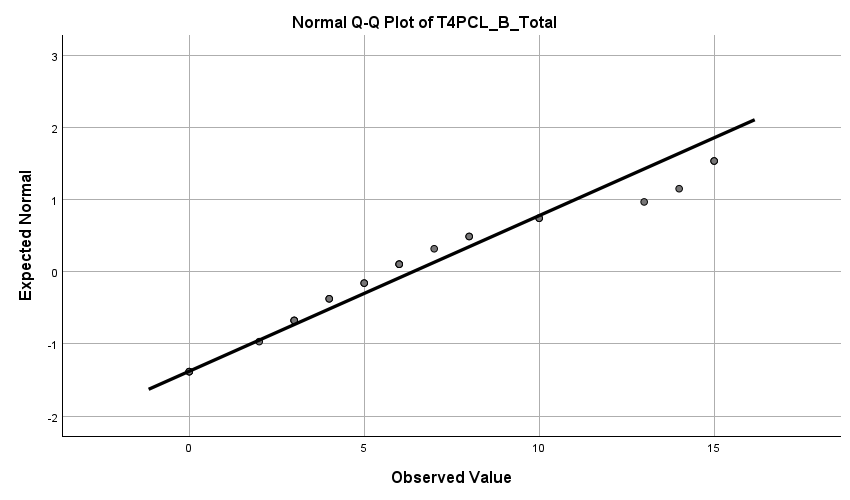
*

*T4 PTSD-A Q-Q Plot*

*
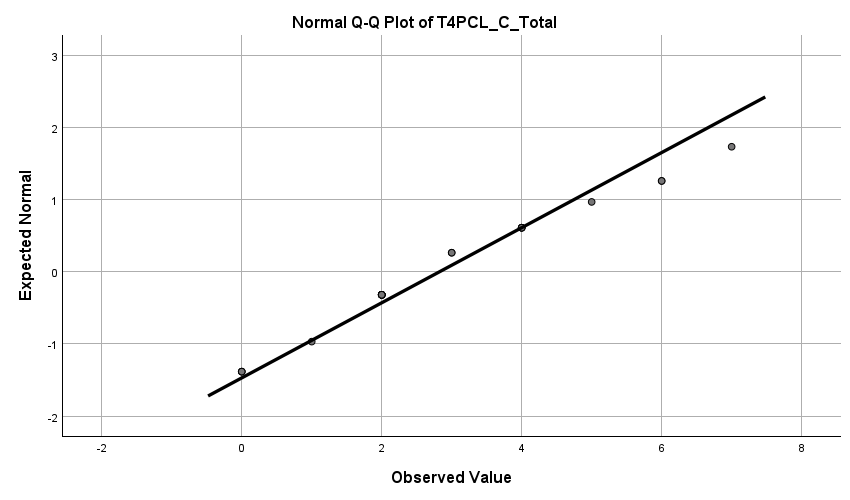
*

*T4 PTSD-N Q-Q Plot*

*
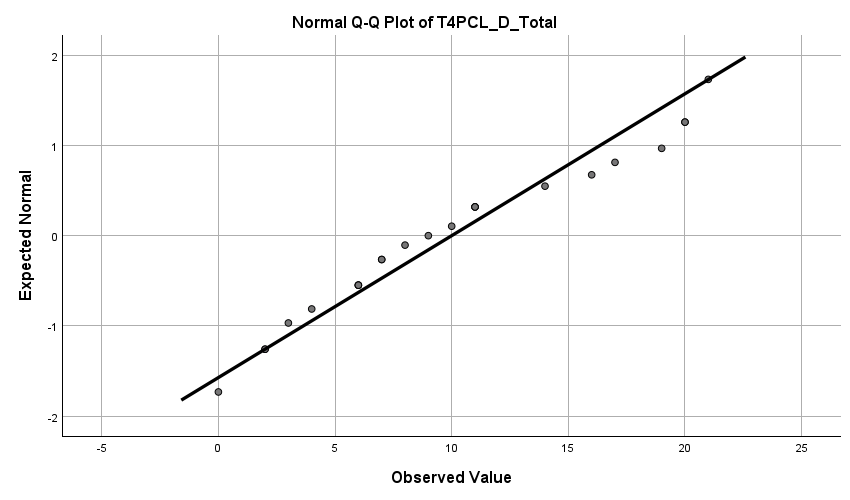
*

*T4 PTSD-H Q-Q Plot*

*
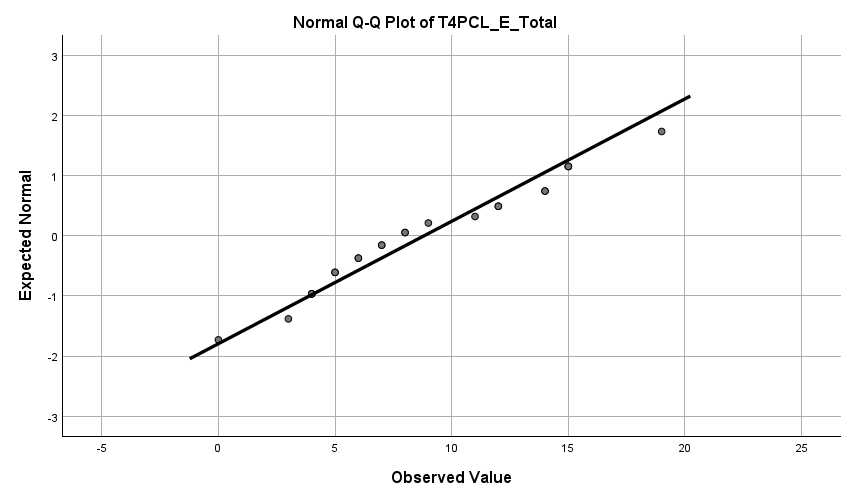
*

*T4 Depression Q-Q Plot*

*
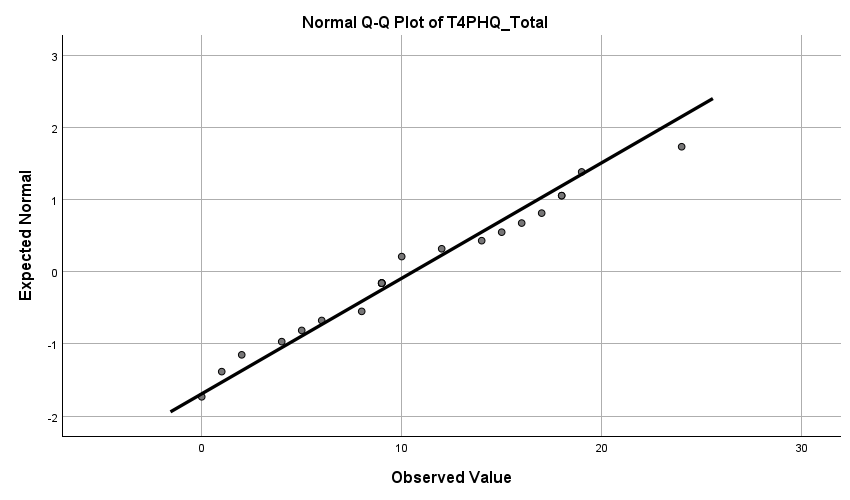
*

*T4 Anxiety Q-Q Plot*

*
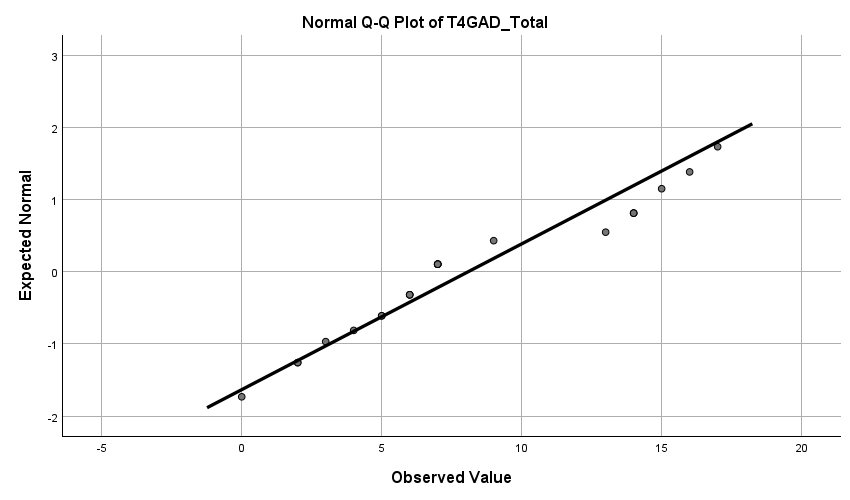
*

*T4 Mindfulness Q-Q Plot*


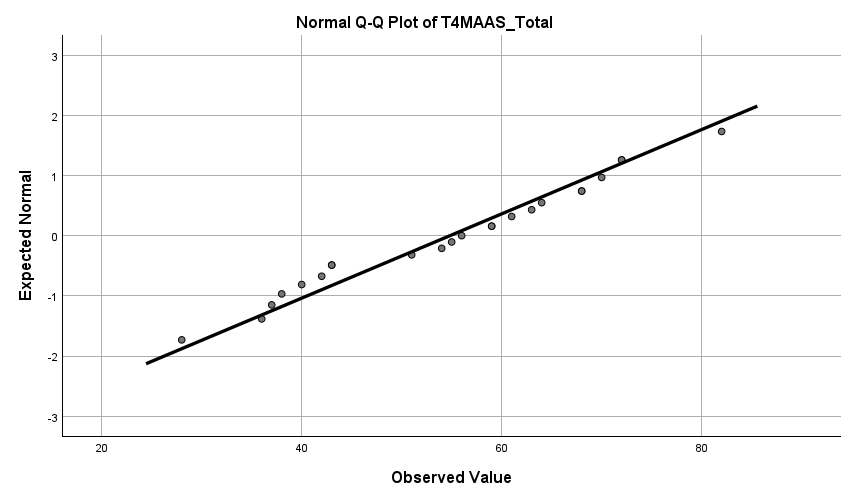

Supplement: usaf393_Supplementary_Data [file usaf393_supplementary_data.docx]
